# Increasing use of nephron sparing surgery for T1a renal tumors

**Published:** 2007

**Authors:** Rajeev Kumar

**Affiliations:** Department of Urology, All India Institute of Medical Sciences, New Delhi, India. E-mail: rajeev02@gmail.com

## SUMMARY

In order to test the hypothesis that discordantly large numbers of laparaoscopic radical nephrectomies were being performed for small renal tumors where nephron-sparing surgery may have been feasible, the authors performed a retrospective review of 381 patients who underwent surgical procedures at their institution for renal cortical tumors during a six-year period beginning January 1998. The aim of the review was to ascertain the appropriateness of the surgical procedure performed with respect to the tumor size and biology. Three hundred and thirty-six patients had an organ-confined tumor. Surgical procedures performed included radical or partial nephrectomy, both open and laparoscopic. The review period was divided into two equal three-year intervals in order to assess any change in trends. Complication data was also evaluated.

There was an increase in the percentage of patients presenting with localized tumors, most prominent for patients with T1a tumors, during the two periods. The authors noted no increase in the number of laparoscopic radical nephrectomies performed for such tumors (20% in both periods). On the other hand, the number of open radical nephrectomies decreased from 23 to 9% while the number of partial nephrectomies increased from 56 to 71%. The authors also noted a shift in the indications for nephron-sparing surgery with elective procedures being performed in 40% patients in the later period compared with 28% in the earlier period. The authors concluded that despite the increased availability of laparoscopic techniques, small renal tumors continue to be appropriately treated with nephron-sparing surgery.

## COMMENTS

This is a timely and well written manuscript addressing ethical concerns about the misuse of technology. Minimal access surgery has become one of the hallmarks of progress in most surgical specialties and in urology in particular. The advantages of laparoscopy are well documented and its acceptance or rather demand, by patients has been mirrored by its availability at increasing number of centers. This has been coupled with the view that open surgery is a choice of last resort and is to be rarely utilized.

Nephron-sparing surgery for T1a renal tumors has been shown to have both short- and long-term results equivalent to those of radical nephrectomy.[1] It is also established that while the single remnant kidney after nephrectomy for transplant is sufficient to maintain renal function, decreasing renal mass may damage the remnant nephrons.[2] There is also a concern about tumor recurrence and it simply seems logical that if removing half a kidney is good enough and safe, why remove the whole? However, nephron-sparing surgery is a more difficult procedure compared with radical nephrectomy. This difference in technical difficulty is enhanced in laparoscopy and there are probably only a handful of surgeons routinely performing laparoscopic partial nephrectomy. In the face of increasing numbers of small tumors being detected through imaging for unrelated conditions, the dichotomy between demand and supply would lead to a reasonable assumption that laparoscopic radical nephrectomies are being inappropriately performed for tumors amenable to nephron-sparing surgery.

This concern was further heightened by a number of recent reviews. The SEER database was reviewed by authors from the same group as this manuscript and they found that only 42% patients with tumors less than 2 cm underwent partial nephrectomy in 2000-2001 and this number decreased to 20% in those with tumors between 2 to 4 cm.[3] The SEER database is considered representative of the entire US population. Fortunately, the trend towards utilization of partial nephrectomy showed a progressive increase over the years reviewed. Another review of the Nationwide Inpatient Sample found that nearly 90% of all surgeries for renal cancer were radical nephrectomies.[4] While this database did not have details about the tumor stage or surgical technique, the numbers seem much larger than the actual frequency of tumors greater than 4 cm.

The current study is reassuring that despite the seemingly increasing use of laparoscopic radical nephrectomy, the majority of patients with small renal tumors get the partial nephrectomy that they deserve. Unfortunately, this data comes from an institution with surgeons skilled in the art of both open and laparoscopic partial nephrectomy and who, due to the academic nature of their institution, are probably more prone to scrutiny of their surgical decision-making than a single-surgeon practice.
